# Pervasive and opposing effects of Unpredictable Chronic Mild Stress (UCMS) on hippocampal gene expression in BALB/cJ and C57BL/6J mouse strains

**DOI:** 10.1186/s12864-015-1431-6

**Published:** 2015-04-03

**Authors:** Karim Malki, Yann S Mineur, Maria Grazia Tosto, James Campbell, Priya Karia, Irfan Jumabhoy, Frans Sluyter, Wim E Crusio, Leonard C Schalkwyk

**Affiliations:** MRC SGDP Centre, King’s College London at the Institute of Psychiatry, PO80, DeCrespigny Park, London, UK; Present address: Department of Psychiatry, Yale School of Medicine, New Haven, USA; The Institute of Cancer Research, London, UK; Present address: University of Bordeaux, Institute for Cognitive and Integrative Neuroscience (INCIA), Bordeaux, France; Department of Psychology, Tomsk State University, Tomsk, Russia; School of Biological Sciences, University of Essex, Colchester, UK; Brudnick Neuropsychiatric Research Institute, University of Massachusetts Medical School, Worcester, MA 01604, USA USA

**Keywords:** BALB/cJ, UCMS, Unpredictable chronic mild stress, C57BL/6J, Stress, *Ubc*

## Abstract

**Background:**

BALB/cJ is a strain susceptible to stress and extremely susceptible to a defective hedonic impact in response to chronic stressors. The strain offers much promise as an animal model for the study of stress related disorders. We present a comparative hippocampal gene expression study on the effects of unpredictable chronic mild stress on BALB/cJ and C57BL/6J mice. Affymetrix MOE 430 was used to measure hippocampal gene expression from 16 animals of two different strains (BALB/cJ and C57BL/6J) of both sexes and subjected to either unpredictable chronic mild stress (UCMS) or no stress. Differences were statistically evaluated through supervised and unsupervised linear modelling and using Weighted Gene Coexpression Network Analysis (WGCNA). In order to gain further understanding into mechanisms related to stress response, we cross-validated our results with a parallel study from the GENDEP project using WGCNA in a meta-analysis design.

**Results:**

The effects of UCMS are visible through Principal Component Analysis which highlights the stress sensitivity of the BALB/cJ strain. A number of genes and gene networks related to stress response were uncovered including the *Creb1* gene. WGCNA and pathway analysis revealed a gene network centered on *Nfkb1*. Results from the meta-analysis revealed a highly significant gene pathway centred on the Ubiquitin C (*Ubc*) gene. All pathways uncovered are associated with inflammation and immune response.

**Conclusions:**

The study investigated the molecular mechanisms underlying the response to adverse environment in an animal model using a GxE design. Stress-related differences were visible at the genomic level through PCA analysis highlighting the high sensitivity of BALB/cJ animals to environmental stressors. Several candidate genes and gene networks reported are associated with inflammation and neurogenesis and could serve to inform candidate gene selection in human studies and provide additional insight into the pathology of Major Depressive Disorder.

**Electronic supplementary material:**

The online version of this article (doi:10.1186/s12864-015-1431-6) contains supplementary material, which is available to authorized users.

## Background

Major depressive disorder (MDD), characterised by a core of low mood, anhedonia and fatigability, has a lifetime prevalence of 15–20% and is associated with significant morbidity and mortality, particularly from a range of chronic health conditions [1-3]. Research into this complex disorder has traditionally used animal models, which are broadly grouped as either tests of antidepressant action (e.g. forced swim test) or models of depression-like behaviors (e.g. chronic social defeat). The advantages of using animal models including the ability to control for environmental factors and the access to post-mortem brain tissue are balanced by inherent limitations, most notably the inability to convincingly replicate several symptoms (e.g. suicidal ideation and delusions) involved in the clinical diagnosis of MDD [4]. In addition, given our lack of understanding of causative agents in MDD, and the multitude of possible factors, the majority of these models lack aetiological validity.

This is overcome, in part, by the use of stressor-based models as exposure to stressful life events has previously been implicated in MDD [5-9]. Unpredictable chronic mild stress (UCMS) involves subjecting rodents to a series of repeated physical stressors (e.g. food and water deprivation, cage tilting, social isolation etc.) for a prolonged period of time (usually over 2 weeks) [10-12]. This results in the development of anhedonic features (e.g. reduced sucrose intake) that are sensitive to chronic, but not acute administration of antidepressants, and thus more closely resembles the chronic, multidimensional nature of clinical MDD [13,14]. The validity, reliability and limitations of UCMS protocols have been discussed extensively [15]. The value of such a mouse model is further enhanced by the use of inbred mice strains, which allow the production of multiple progeny with stable and identical genetic material, but with differing phenotypic characteristics. For instance, it has been shown that inbred strains of mice present different thresholds of susceptibility to UCMS [16-19].

Two commonly used strains are C57BL/6J and BALB/cJ, which differ in several regards. Principally, BALB/cJ appears to exhibit markedly elevated levels of anxiety and stress reactivity, compared with the relatively nonanxious C57BL/6J strain, across several different approach/avoidance paradigms [20-25]. These mice also display altered stress-related behaviours such as stretch-attend postures, defecation, and licking/grooming, with a corresponding increase in activation of the hypothalamic-pituitary-adrenal (HPA) axis - a regulator of the stress response [26-29]. Conversely, studies have indicated that C57BL/6J mice demonstrate greater depression-like behaviour in several paradigms, including the forced swim test [30-33]. Furthermore, BALB/cJ, but not C57BL/6J mice, were found to be sensitive to chronic, but not sub-chronic/daily, antidepressant administration [21,34].

The behavioural variation found between strains of inbred mice represent potentially valuable information on the molecular mechanisms underlying MDD, and can be investigated using gene expression profiling across strains [35]. Of particular interest is the hippocampal brain region; a main target for the action of glucocorticoid stress hormones which underlie cognitive performance, behavioural adaptation and regulation of the HPA-axis. Further, direct regulation of cholinergic signaling in this brain area can induce depression-like phenotypes and is also critical for antidepressant efficacy [36,37]. In MDD, chronic administration of different classes of antidepressants increases the proliferation, as well as the number, of newly formed neurones in this area, an effect not seen with non-antidepressant psychotropic medications [38].

In the present experiment we assessed hippocampal gene expression profiles across two inbred strains of mice subjected to either UCMS or no treatment in order to examine the potential effects of UCMS on gene expression.

## Methods

### Design

We explored mRNA expression differences in response to Unpredictable Chronic Mild Stress (UCMS) in two inbred mouse strains (BALB/cJ and C57BL/6J). An equal number of male and female mice from each strain were used in a balanced design. Half the animals from each strain were assigned to the UCMS group and the other half to a Control group. The study consisted of 2 strains (BALB/cJ, C57BL/6J), 2 two sexes (Male and Female), and two stress conditions (UCMS, Control) for a total of 8 experimental cells. In order to gain further insight into the molecular mechanisms that may play a role in stress response, we cross-validated gene modules associated with stress response with a parallel study from the GENDEP project. This was done using WGCNA (Weighted Gene Co-Expression Network analysis) in a meta-analysis design. A detailed description of the GENDEP mouse study is found elsewhere [39,40]). Briefly, the GENDEP study design consisted of 4 inbred mouse strains (129S1/SvlmJ, C57LB/6J, DBA/2J and FVB/NJ) exposed to two depressogenic protocols (Unpredictable Chronic Mild Stress and Maternal Separation) and a Control condition.

### Animals

Breeders were purchased from The Jackson Laboratory (Bar Harbor, ME, USA) and bred at the animal facility at UMass Medical School, Worcester, for at least two generations. Weaning took place at 28 ±1 days and mice from 3 to 7 different litters per strain and treatment condition were housed in same sex groups of 2 to 4 litter mates until they reached the age of 2 months ±1 week. At this age, animals were ear-punched for identification and assigned to one of two treatment groups, controls and UCMS. The UCMS treatment started at the same time and continued during the behavioural testing phase. However, care was taken not to apply stressors just before a test. The tests took place 7 weeks after the beginning of the stress procedure. Due to the nature of the UCMS procedure, stressed and control animals were kept in separate but otherwise identical holding rooms. Breeding, UCMS protocol and micro-dissections were done on-site at the University of Massachusetts. RNA extraction from frozen hippocampi samples and microarray analysis was performed at the MRC SGDP center, at the Institute of Psychiatry, London. Animals from the GENDEP study used for the WGCNA meta-analysis have been described in more detail elsewhere [41-43]. All housing and experimental procedures for the GENDEP study were carried out in accordance with the UK Home Office Animals (Scientific Procedures) Act, 1986.

### Unpredictable Chronic Mild Stress UCMS

The UCMS model is based on repeated and inescapable stressors that eventually lead to depression-like phenotypes in mice. This approach stems from previous studies showing that stress and major life events are critical risks factors for major depressive disorders [44]. Mice were subjected to different kinds of stressors such as: cage tilting, damp sawdust, predator sounds, placement in an empty cage, placement in an empty cage with water on the bottom, inversion of the light/dark cycle, lights on for a short period of time during the dark phase, and switching cages following previously published protocols [41,45]. On average, two of these stressors were applied daily following a semi-random two-week schedule. The stress procedure lasted for 4 weeks prior to the behavioural testing. Stressors continued to be applied during the testing phase, except on testing days to avoid effects of acute stress. At least 12 hours of rest was provided between a stressor and a test. The stress procedure did not include any food or water deprivation.

### mRNA extraction

Total RNA was extracted using the guanidium thiocyanate method with TRIzol (Invitrogen Life Technologies, UK) from 16 hippocampi (RNA preparation); (2) mRNA converted to cDNA using reverse transcriptase and poly-T primer; (3) Resulting cDNA was amplified using T7 RNA polymerase in the presence of biotin-UTP and biotin-CTP, so each cDNA will yield 50–100 copies of biotin-labeles cRNA; (4) cRNA was incubated at 94° in fragmentation buffer to produce cRNA fragments of length 35 to 200 nucleotides; (b) Fragmented cRNA was hybridized to the GeneChip Mouse Genome 430A 2.0 (c) scanned in laser scanner and (d) data analysis performed. Brains were dissected and hippocampi frozen on dry ice. The Affymetrix GeneChip Mouse Genome 430 2.0(c) array was chosen because at the time of the experiment, it provided the most comprehensive coverage of the mouse genome. These high-density arrays contain multiple probe pairs per probe set, providing several independent measurements for every transcript. The array contains 45101 probe sets that measure the expression level of more than 39,000 transcripts and variants, covering in excess of 19,000 well-characterized mouse genes.

## Statistical analysis

Mean probe set intensities for 16 mice (eight of strain C57BL/6J and eight of strain BALB/cJ) from Affymetrix MOE 430 arrays were normalized and summarized using Robust Multichip Average (RMA) method [46]. Probe sets that were systematically absent (based on the MAS 5.0 detection present/absent call) across all the arrays were removed leaving 22,690 probe sets [47]. Prior to analysis, a plot of ordered probe set intensities was produced to establish a suitable threshold above which intensities could be considered true signals. The threshold chosen was a mean probe set intensity of 250 that resulted in the inclusion of 8627 measurements.

### Principal Component Analysis/Partial Least Squares (PCA/PLS)

Prior to PCA, probe-sets were further processed by using mean-centering and Pareto scaling. Pareto scaling uses the square root of the standard deviation as the scaling factor. This method was chosen because it has two advantages compared to traditional methods such as autoscaling. First, large fold changes are penalized more than small fold changes. Second the data does not become dimensionless as with other scaling methods [48]. Principal component Analysis (PCA) was performed on the data using processed probe sets as X -variables. Two new Y-variables were added to the data encoding: one pertaining to strain (BALB/cJ or C57BL/6J) and one to the stress treatment (UCMS or control). PCA was used to assess structure in the mouse transcript data set and to summarize the data set in an unbiased manner. This enables any potential issues, such as unusual observations, to be investigated prior to application of a supervised method. Additionally, it allows us to ascertain if UCMS effects are visible at the genomic level. We then conducted Partial Least Square (PLS) analysis implemented in the SIMCA-P environment (http://www.umetrics.com).

### General Linear Models (GLM)

Main effects and interaction effects relating to strain, sex and stress differences were investigated using general linear models implement in R (http://cran.r-project.org) considering strain, sex and stress as fixed effects and untransformed probe set summaries as dependent variables. In order to identify probe sets that could be involved with the stress-sensitive phenotype of BALB/cJ mice we further performed a correlational analysis in R. Unstressed BALB/cJ animals were coded as group 1 and all remaining animals as group 0. We calculated Pearson’s correlation coefficient (R) between the two groups for each probe set. Highly correlated probe sets, using a |*R*|>0.8 cut-off, were selected and carried forward for pathway analysis using Ingenuity Pathway Analysis (IPA) software (Ingenuity Systems, Inc, Redwood City, California, USA, http://www.ingenuity.com). IPA has been extensively used to obtain networks revealing the molecular association between the genes presented. Each network revealed by IPA is scored according to the fit of significant genes in each dataset using the Fisher exact test.

### Weighted Gene Coexpression Network Analysis (WGCNA) on BALB/cJ and C57BL/6J

WGCNA is designed to identify correlated gene modules, relate them to one another and associate them with experimental factors [49]. WGCNA assigns a connection weight between pairs of genes within the network based on biologically motivated criteria [50]. The identification of relevant modules is achieved by the application of soft threshold to correlations between pairs of genes within a given network. The advantage of this method is that it offers an unsupervised hypothesis-free network-based gene screening to select probe sets associated with a trait that makes it particularly suitable for multifactorial mRNA studies. Prior to module detection, we conducted a sample-clustering to check for the presence of outliers. No outliers were detected and we proceeded with the optimisation of the soft-threshold values. Unassigned co-expression networks were constructed using the blockwiseModule function in the WGCNA package for R [51]. The function first computes a pairwise correlation matrix for each set of genes and then calculates an adjacency matrix by raising the matrix to a soft-threshold power. Decision of the threshold power has been determined using a scale-free topology criterion. Based on the scale of independence we selected a threshold of 5. Probe sets belonging to gene modules associated with stress response were uploaded on the ingenuity pathway analysis software to gain further insight into pathways that may be relevant to stress response.

### Cross-validation and WGCNA Meta-Analysis with GENDEP study

Lastly, we looked for consensus modules between the BALB/cJ and C57BL/6J study and an independent mouse study from the GENDEP project using Weighted Gene Coexpression Network Analysis in a Metanalysis design (43,44). Comparison of coexpression analysis between two independent studies may provide additional insight into stress-related mechanisms. Briefly, data from 144 Affymetrix MOE 430 v2 mouse whole-genome microaarrays was normalized and summarized into probe sets by Robust Multichip Average (RMA) method (37). Additional information on the GENDEP mRNA data preprocessing can be found elsewhere [52]. Probsets from the two versions of the same family of Affymetrix arrays, 430 MOE and 430MOE V2 respectively used in the two studies, were matched using scripts written in Python (https://www.python.org/). We first compared average gene-expression and overall connectivity between the two data sets and these showed high correlation, suggesting similarity across the two datasets (Additional file 1: Figure S1). We then conducted WGCNA adjusting power functions parameters to create topological overlap (TO) as described previously for each of the two studies separately. Modules relating to stress factors where obtained by hierarchical clustering. We then assessed the extent to which modules in the BALB/cJ C57BL/6J study were preserved in the GENDEP study. The module labels from the BALB/cJ/C57BL/6J study grouped in the GENDEP dataset revealed good module preservation (Additional file 2: Figure S2). This was tested using z-scores with the modulePreservation function in the WGCNA package for R, where |*Z*|>10 represents high preservation. Lastly, we compared the two gene-networks for consensus modules to uncover transcriptional similarities across the two studies [53].

## Results

### PCA/PLS

**Figure 1:**
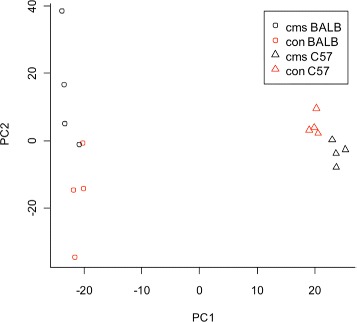
Principal components analysis. Plotting the scores following Pareto scaling shows that the first unrotated principal component of variance can be interpreted as mouse strain. There is a wide separation between C57BL/6J (triangles) and BALB/cJ (circles) but this is expected as different strains reflect a different genetic panel. A compelling result is the separation of samples by treatment in the second component. There are striking differences between the two strains in response to UCMS at this is clearly visible at the genomic level. Additionally, the results show that the direction of the UCMS effect is in opposing direction in C57BL/6J and BALB/6.

**Figure 2:**
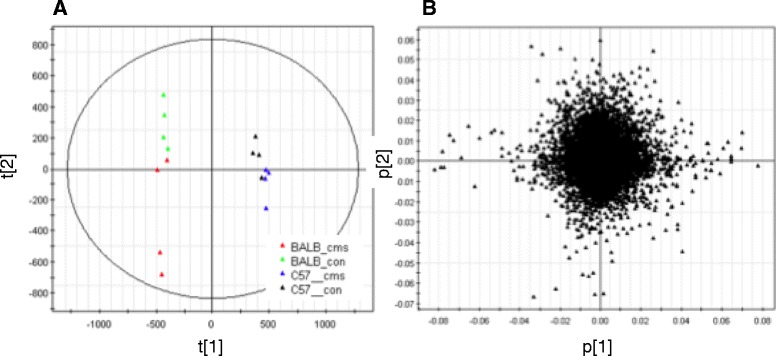
Scores and loading plots. PCA scores and loadings plots for the first two components of a model fitted to the mouse transcript data set. Figure **A** shows scores plot with the observations color coded according the legend. Figure **B** shows the loadings plot of variables.

Principal Component Analysis (PCA) was first used to assess the structure in the mouse mRNA data set (Figure 1). A total of four principal components were fitted to the data set with a total variance explained (*R*^2^) of 0.67 and a cumulative predictive ability (Q2[cum]) of 0.24. Plotting the scores reveals that the first unrotated principal component of variance can be interpreted as mouse strain: there is a wide separation between C57BL/6J (triangles) and BALB/cJ (circles) Figure 2. This is expected as there are numerous known genetically-driven strain differences. More interestingly, there is a clear separation of samples by treatment in the second component (t[2]). This clearly highlights the stress-sensitivity of the BALB/cJ strain. Examination of the loadings enables the interpretation of which probe sets are contributing most to the selected components. The probe sets represented by points at the extreme left and right side of the loadings plot will tend to have greater values in the BALB/cJ and C57BL/6J strain animals, respectively. The probe sets represented by points at the extreme upper and lower points of the loadings plot tend to have greater values in the control and UCMS treated animals, respectively. We then conducted PLS analysis on the same sample. A PLS model was fitted to the mouse transcript data set with four components, of which the first two captured information relating directly to the strains and treatments. The variance explained by the first two PLS components in the X and Y data blocks were 0.44 and 0.90, respectively. The Q2[cum] for the two model components was 0.75. Figure 2 shows plots of the scores and weights for the two PLS components, coloured according to the strain and treatment data (Additional file 3: Figure S3).

As was seen in the PCA scores plot, there is strong separation between the samples derived from the different strains in the first components scores (t[1]) and between the different treatments in the second components scores (t[2]) (Figure 2A). Interpretation of the scores plot can be made through inspection of the weights plot (Figure 2B). PLS coefficients were used to determine the relevance of the probe set intensities to the strain and treatment differences. An ordered plot of the coefficients for the first and second components was used to determine thresholds for selecting a probe set as relevant or not. Thresholds were chosen as absolute coefficient values exceeding 5E-05 for the strain and treatment y-variables in the first and second PLS components, respectively. In each case, the sign associated with the coefficients was interpreted as positive values meaning the probe intensities tended to be higher in C57BL/6J strain and mice exposed to the UCMS protocol, whilst negative values meant that the probe intensities tended to be higher in BALB/cJ strain and control treated mice.

We then explored the number of main effects and interactions as part of a 2×2 ANOVA implemented in R. A summary of probe sets uncovered at a p-value of 0.05, False Discovery Rate and using Bonferroni correction is provided in Table 1. When exploring main effect of stress, two probe sets survived Bonferroni correction while 5 probe sets in the stress X strain interaction analysis survived the same correction method. The differences by treatment and the interaction, even at the Bonferroni level shows that the UCMS stress protocol causes a sufficiently strong biological insult in the stress-responsive BALB/cJ. Probe sets were subsequently annotated with Panther (www.panther.com); a summary of the top interactions and UCMS main effect hits at Bonferroni adjusted level of significance is found in Table 2.

**Table 1:** **The results from a 2X2 ANOVA for each of the 22690 probesets on the array gives the following numbers of candidates at the 0.05 cut off levels**

	**Nominal**	**FDR**	**Bonferrorni**
Environment	3163	22	2
Strain	6435	4742	555
Interaction	4274	626	5

**Table 2:** **The table shows the probe sets and corresponding gene name uncovered from the ANOVA analysis**

**Top interaction hits**		
**Probe set**	**Gene name**	**Description**
1420093_s_at	*Hnrpdl*	heterogeneous nuclear
		ribonucleoprotein D-like
1424891_a_at	*Zw10*	ZW10 homolog (Drosophila),
		centromere/kinetochore protein
1448609_at	*Tst*	thiosulfate sulfurtransferase,
		mitochondrial
1449039_a_at	*Hnrpdl*	heterogeneous nuclear
		ribonucleoprotein D-like
1450779_at	*Fabp7*	fatty acid binding protein 7, brain
**Top UCMS hits**		
1433439_at	*Cpne1*	copine I
1448609_at	*Tst*	thiosulfate sulfurtransferase,
		mitochondrial

All the probes sets listed survived Bonferroni correction for the number of multiple non-independent testing.

**Figure 3:**
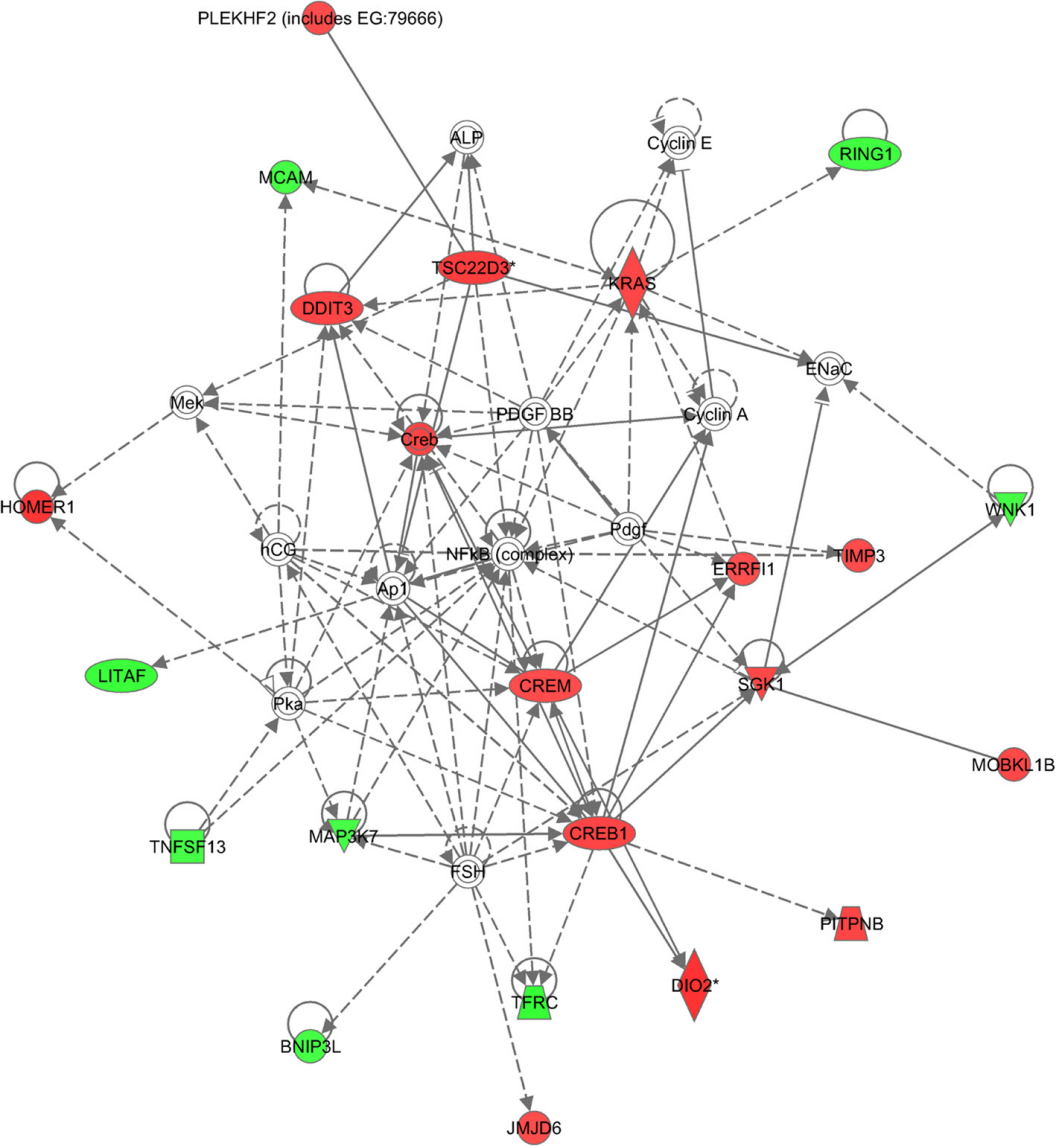
IPA Network. 119 unique probe sets were obtained through correlational analysis by coding UCMS BALB/cJ animals as group one and all other animals as group 2 at the stringent |*R*|>0.8 cut-off level. The results were uploaded on Ingenuity Pathway Analysis and a significant network centred on the *Nfkb* gene hub was returned. The *Nfkb* has been associated with proinflammatory cytokines, such as IL-1beta which have been implicated in the cellular and behavioral effects of stress response and in mood disorders. Regulation of *Nfkb* has further been suggested to modulate hippocampal neurogenesis. Nodes on the diagram color coded represent reference molecules, with red indicating a positive correlation value and green indicating a negative correlation value.

In order to cast a wider net, the 626 interaction hits at q(FDR) =0.05 were uploaded to the Ingenuity Pathway Analysis software. IPA revealed a group of 11 behaviourally interesting genes (Table 3). Of particular interest are the three probesets that are high in the BALB-UCMS group and essentially constant in the other groups (Creb 1452529_a_at, Crem 1418322__at, Homer1 1425671_ at), which could be involved with the stress-sensitive phenotype of BALB/cJ mice. To look for transcripts following this pattern, we created two groups: BALB-UCMS group as group one and the remaining animals as group two and calculated pearson’s R for this variable with each probe set. The three probe sets mentioned above show correlations of 0.85, 0.82, and 0.92 respectively. At the stringent cut-off of |*R*|>0.8, nominal p-value of 2e-5 and q-value <0.026, we obtain 119 probe sets. Probe sets were uploaded to the Ingenuity Pathway Analysis software. IPA returned a gene network comprising 23 reference molecules with a score >40. 15 genes belonging to this network were positively correlated with BALB/cJ while 8 were negatively correlated. Nodes in colour are on the |*R*|>0.8 list, red are positively correlated (ie BALB/cJ high) and green are negatively correlated. The pathway returned is of particular interest as it is centred on the Nfkb complex (Figure 3). Nuclear factor *Nfkb*, is relevant to the depression in two ways. First it has been shown to modulate glucocorticoid receptor signalling and thereby regulating CRH and proinflammatory cytokins release. This is congruent with the inflammation hypothesis of MDD. Second it plays a role in neuronal cell growth, survival and synaptic plasticity, which makes it relevant to the neurogenesis hypothesis of MDD [38].

**Table 3:** **The 626 interaction hits from the ANOVA model using strain and stress as main effects, at the c threshold of q = 0.05 were uploaded on the Ingenuity Pathway Analysis database**

**Symbol**	**Entrez gene name**	**Affymetrix**	**Location**	**Type**
*Creb1*	cAMP responsive element binding protein 1	1452529_a_at	Nucleus	transcription regulator
*Crem*	cAMP responsive element modulator	1418322_at	Nucleus	transcription regulator
*Fyn*	FYN oncogene related to SRC, FGR, YES	1448765_at	Plasma Membrane	kinase
*Grik1*	glutamate receptor, ionotropic, kainate 1	1427676_a_at	Plasma Membrane	ion channel
*Homer1*	homer homolog 1 (Drosophila)	1425671_at	Plasma Membrane	other
*Htr1a*	5-hydroxytryptamine (serotonin) receptor 1A	1450219_at	Plasma Membrane	G-protein coupled receptor
*Il1b*	interleukin 1, beta	1449399_a_at	Extracellular Space	cytokine
*Ncam1*	neural cell adhesion molecule 1	1421966_at	Plasma Membrane	other
*Npy*	neuropeptide Y	1419127_at	Extracellular Space	other
*Ptpra*	protein tyrosine phosphatase, receptor type, A	1425340_a_at	Plasma Membrane	phosphatase
*Vldlr*	very low density lipoprotein receptor	1417900_a_at	Plasma Membrane	transporter

IPA revealed a group of 11 associated genes associated with a stress response phenotype, several of which have been previously associated with depression and depression-related phenotypes.

### Weighted gene co-expression network analysis

**Figure 4:**
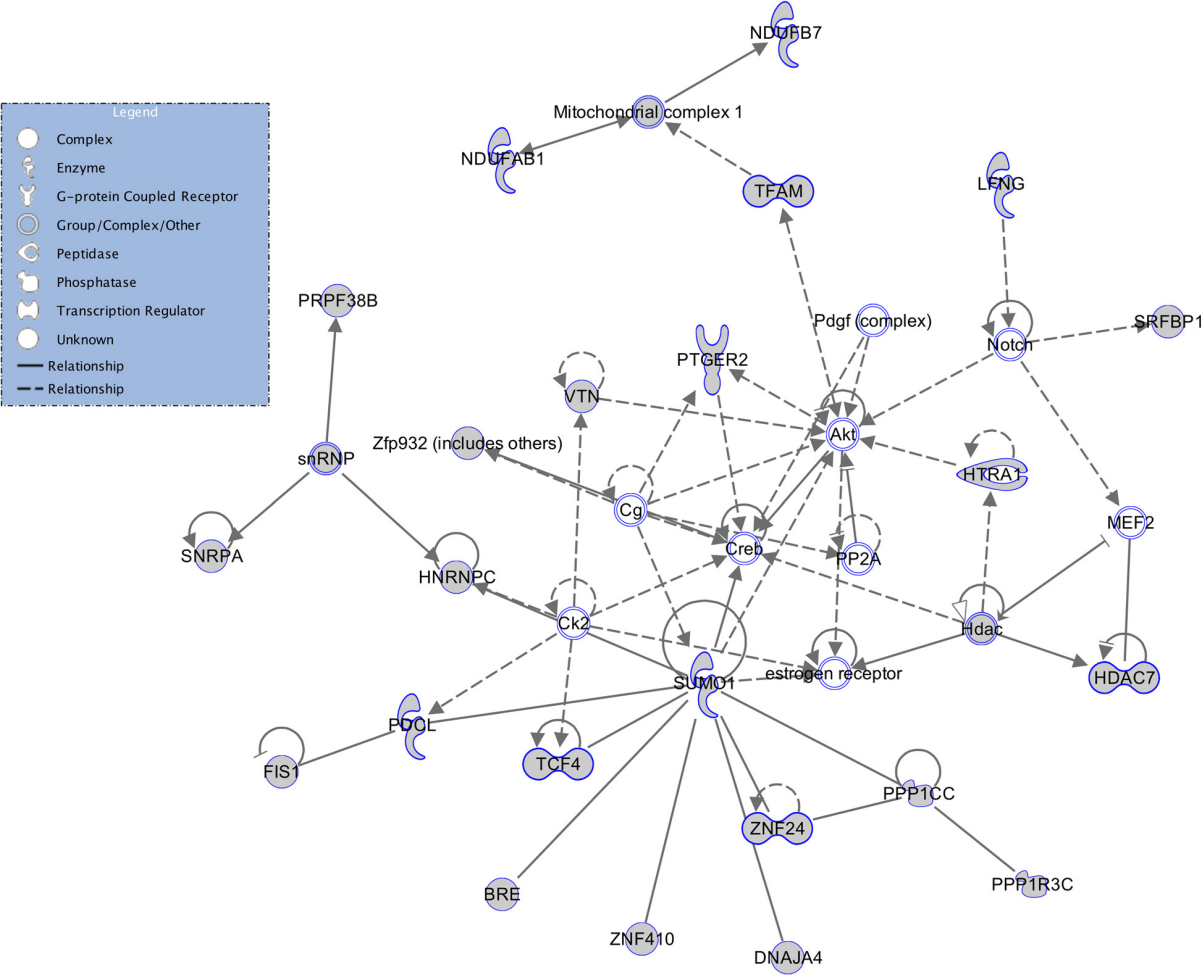
IPA from WGCNA results. Results from Weighted Gene Coexpression Network Analysis revaled 858 coordinatly expressed probes associated with stress factors. The results were uploaded on the Ingenuity database and a signficant network, centred on the *Creb1* gene hub was returned. The *Creb* gene has extensively been associated with depression-related phenotypes as well as antidepressant treatment response.

WGCNA was used to explore the functional organization of the transcriptome in response to UCMS by constructing a weighted gene coexpression network. WGCNA uses the topological overlap (TO) to measure the coexpression profile of genes pairs and neighbouring genes across the network to uncover patterns of coexpression across the transciptome. A total of 31 gene modules with at least 100 genes per modules were returned based on hierarchical clustering. Gene modules were then subsequently related to the stress and strain factors. Four gene modules in each condition were nominally associated with the two experimental factors (stress and strain). Several modules are significantly associated with strain effects but these are expected as strain differences reflect a different genetic background. One module showed a significant correlation with stress and survives Bonferroni correction for the number of modules tested *R*=0.64, *p*=0.007. This module contains a total of 858 coordinately expressed probe sets potentially involved in stress response mechanism. These are subsequently uploaded to the Ingenuity Pathway Analysis database. The results from IPA reveal a significant stress-related gene network centred on the *Creb1* gene hub (Figure 4). The *Creb1* gene has long been nominated as potential convergent target for a wide-range of antidepressant drugs and several animal studies have reported an association between *Creb1* and depressive-related phenotypes [54].

### Cross validation using WGCNA meta-analysis

**Figure 5:**
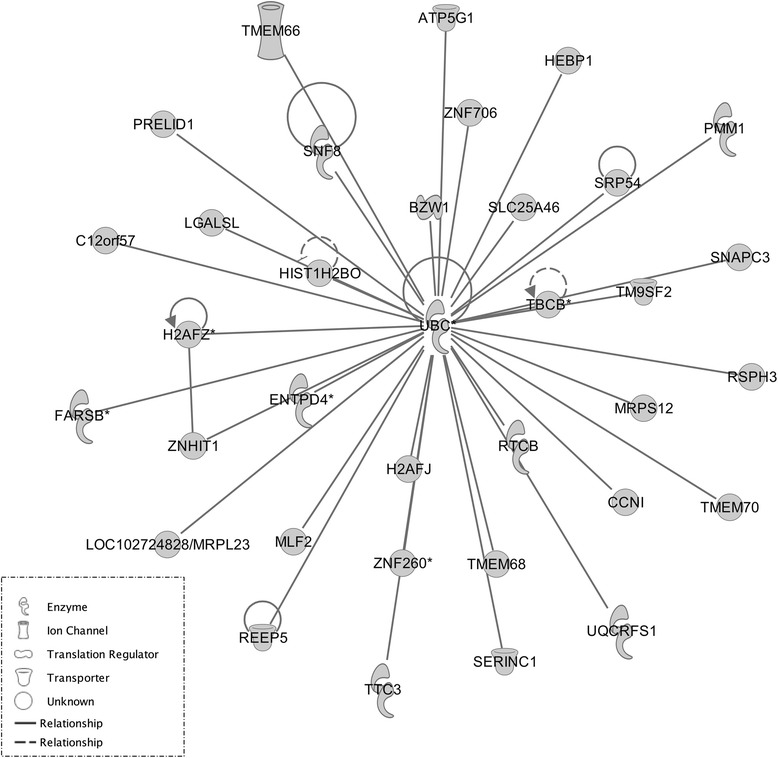
IPA from WGCNA Meta analysis results with the GENDEP project. In order to cross-validate the significant module from the WGCNA analysis and gain additional insight into molecular mechanism associated with stress, related phenotypes, the results from convergent gene-modules associated with stress response across the two, independent studies obtained with WGCNA meta-analysis were uploaded on IPA. IPA returned a highly scoring gene network clearly centered on the Ubiquitun-C (*UBC*) gene. Ubiquitin-C has been previously associated with mood disorders and it is also known to be a stress-inducible protein.

As there is generally extensive variability across microarray studies, it is important to cross-validate results in an independent dataset. We have chosen a parallel investigation from the GENDEP mouse arm that used similar UCMS stress regime across 4 inbred mouse strains. We used Weighted Gene CoExpression Network Analysis to compare two networks as described by Miller et al. [53,55]. We measured the correlation between each gene and Module Membership using kME values. This resulted in 17 gene-modules being significantly correlated across the two studies. We then determined which genes are hubs across both networks by selecting genes within each module correlating with stress response with the highest kME values. A total of 170 probe-sets were uncovered by this analysis and results were uploaded on IPA Software. Out of 170 probe sets uploaded, 169 were found on the Ingenuity Pathway Analysis reference database. The results returned a gene network centred on the UBC, (Ubiquitin C) hub with a compelling score of 82 and consisting of 35 reference molecules (Figure 5). The gene-network uncovered is functionally plausible and the association between *Ubc* and stress response, well documented. The results from the Consensus modules across the two experiments may help to point at putative mechanisms with higher probability of being involved in stress response.

## Discussion

This study investigated gene expression differences in the context of a GxE model using two contrasting mouse strains, BALB/cJ, a highly stress-reactive, anxious and emotive strain with low basal locomotor activity and C57BL/6, which is considered a less emotive and anxious strain [56-59]. Additionally, C57BL/6J mice have been shown to produce twice the amount of serotonin in the forebrain compared to BALB/cJ mice [60]. BALB/cJ mice are known to exhibit depressive-related behaviours when subjected to selected stress paradigms and offer much promise for the study of stress response and as model of depression and antidepressant treatment response in humans.

One of the more compelling findings from our study is the ability to separate groups by stress as well as strain, simply through Principal Component Analysis. The separation by stress at the genomic level clearly points at a sparsely distributed gene expression signal, sufficiently pervasive to be clearly detected by PCA. Additionally, the directionality of the effect seems to be in the opposite direction when compared to C57BL/6J. This highlights the stress-sensitivity of the BALB/cJ mice and can be used to identify gene and gene networks differentially regulated in response to a chronic environmental stressor. To gain further understanding into mechanisms underpinning this effect, we used a correlational design to uncover genes with higher prior probability of association to stress response. Pathway analysis on the genes that most highly correlate with genomic stress differences belonged to a network centered on the *Nfkb* (nuclear factor kappa B) gene complex, which plays an important role in the regulation of oxidative stress-induced cell activation.

The *Nfkb* gene complex can be stimulated through environmental stressors and is considered a prototypical proinflammatory signalling pathway. This pathway has been extensively associated with depressive related disorders and is consistent with the inflammation hypothesis of MDD. *Nfkb* is activated by proinflammatory cytokines including interleukin 1 (IL-1) and tumor necrosis factor (TNF *α*). Moreover, this gene complex has been shown to play a role the expression of other proinflammatory genes, cytokines, chemokines, and adhesion molecules and plays an important role in modulating proinflammatory cytokine production, leukocyte recruitment, and cell survival [61].

We further explored the correlational patterns among genes across microarray samples using Weighted Gene Coexpression Network Analysis WGCNA. WGCNA has proven successful as an unsupervised method to identify clusters of highly coexpressed genes and relating them to external traits. Network pathway analysis conducted on modules of coexpressed genes significantly associated with stress response, returned a network centered on the *Creb1* gene complex. The transcription factor cAMP response element-binding protein *Creb1* is of particular interest and has been extensively implicated in both the pathophysiology of MDD as well as in antidepressant treatment response.

*Creb1* is a transcription factor that regulates a number of different cellular responses including cell proliferation, cell survival and plasticity and differentiation. Indeed several studies have shown that administration of cAMP breakdown inhibitors increases adult hippocampal neurogenesis in mice. Conversely hippocampal cell proliferation is decreased in transgenic mice that express a dominant negative mutant of *Creb1* in the hippocampus [62]. Administration of antidepressant treatment has been shown to increase *CREB* hippocampal neurogenesis whilst reversing the negative effects of stress on this brain region.

Additionally, *Creb1* is induced by a range of factors including inflammatory signals. Indeed there is growing literature suggesting that phosphorylated *Creb1* may directly inhibit *Nfkb* through mechanisms that include blocking the binding of CREB proteins to the *Nfkb* complex, increased production of IL-10 and generation of Tregs [63]. The results from both the correlational and coexpression analysis converge towards gene and gene networks implicated in inflammatory signals. Indeed a previous association between the same two mouse strain and peripheral cytokins has been reported by Palumbo et al., [64]. Their results suggest that stress induces a Th2 response in BALB/cJ and a Th1 response in C57BL/6J mice. Dysregulation in cytokins have been systematically implicated in human pathologies including major depressive disorders and autoimmune diseases [65,66].

In order to gain additional insight into genes and gene networks implicated in stress-response we performed a WGCNA meta analysis with a parallel animal model from the GENDEP project that utilises four inbred mouse strains and the same stress paradigm (UCMS) Intuitively, we hypothesised that there would be convergence of stress-dependent gene modules that may provide additional clues on molecules related to stress effects. The analysis revealed good concordance across the studies. Pathway analysis on convergent genes across both studies associated with stress revealed a highly significant network centred on the Ubiquitin-C (*Ubc*) gene hub.

Ubiquitin-dependent protein degradation is essential for cells to survive a wide-range of environmental stresses. Perturbations in UBC levels could affect cell-survival DNA repair, and regulation of other cell signaling pathways. Ubiquitin can bind to target proteins via an isopeptide bond via different Lys residues of the ubiquitin (polyubiquitin chains). When conjugated with target proteins, polyubiquitin chains serve different functions depending on the Lys residue of the linked ubiquitin. Lys-33-linked and Lys-63-linked ubiquitin plays a role in kinase modification and signalling processes and leads to activation of the transcription factor *Nfkb*. Several studies of the *Nfkb* pathway have shown a role for ubiquitination in addition to its well-known role in targeting protein degradation. Ubiquitin has been associated with different stages in the *Nfkb* pathway including, degradation of the *Nfkb*, processing of *Nfkb* precursors inhibitor Ikb, and activation of the Ikb kinase (IKK) via mechanisms that are independent from those that regulate degradation [67-70]. Moreover several animal studies suggest that the CREB repressor substrate is a target for degradation by the ubiquitin-proteasome pathway [71,72]. Taken in unison, the gene pathways identified across each of the analyses converge towards mechanisms involving inflammation. It has been suggested that the inflammatory response is contained within the stress response as the same pathways are used. Changes in one system can therefore lead to alternations in the other. Regarding the lack of interaction with strain, this suggests that while inflammation is a risk factor for MDD, other interactions are critical for the development of depressive-like symptoms. We have found a similar outcome with neurogenesis, where broad decrease was found across strains but did not correlate with the behavioral outcomes we observed.

An interesting gene affected by UCMS exposure in our study is the *Il1b* gene. *Il1b*, a pro-inflammatory cytokine, has been shown to mediate the antineurogenic effects of UCMS through activation of the *Nfkb* pathway. Inhibiting this pathway pharmacologically or blocking *Il1b* signalling using receptor knockout mice, normalised sucrose preference, a measure of anhedonia as well as other depression and anxiety related behaviours. Studies have shown that this gene plays a role in regularising the proliferation of neural stem-like cells in UCMS exposed mice, highlighting the importance of *Il1b* - *Nfkb* signalling in chronic stress paradigms [73,74]. Our findings support the above studies as well as underline the general role of neuroinflammation in stress disorders and depression.

A further, functionally plausible gene uncovered by our study is the *Npy* gene. This gene has been previously associated with stress and emotional behavior in humans and animals [75]. Interestingly, *Npy* gene is often mentioned in studies on stress resilience, which is attracting increasing attention in studies on depression and PTSD. Resilience is defined as the capacity of an individual to avoid negative social, psychological, and biological consequences of extreme stress that would otherwise compromise their psychological or physical well-being. Resilience can be passive, i.e. the absence of key molecular abnormalities which occur in susceptible individuals, and/or active, i.e. novel molecular adaptations which occur uniquely in resilient individuals to help promote normal behavioural function. *Npy* gene is viewed as part of the latter and, although its precise role in stress coping strategies and depression needs further study, it has been suggested that the *Npy* gene is generally protective [76].

Lastly, we explored genes involved in the strain-stress interaction. Only 5 genes survived Bonferroni correction for multiple, non-independent, tests. However, none of these genes have known association with stress response. Moreover, the molecular substrate underpinning stress-response may be highly polygenic and may not depend on single genes of high penetrance.

The study was successful in highlighting genes and genes network that may be associated with stress response. As hypothesised, BALB/cJ animals showed genomic alterations in response to environmental stressors. The findings presented may be used to inform future human studies on Major Depressive Disorder where accurate history on the onset of stressful life events and access to disease relevant brain tissues cannot be collected. Indeed previous studies have shown that BALB/cJ mice responded to chronic fluoxetine administration following a stress regime, compared with the non-anxious C57BL/6J where the same drug had little effect. This is consistent with equivalent observation in humans, where antidepressants will induce a more potent effect in those with a pre-existing mood imbalance and little effect in individuals who are known to be well. Indeed the environmental stressor plays against a genetic panel that may predispose and individual to develop stress related disorder at some stage in life.

Although we have provided evidence for a role of the genes and gene pathways involved in stress response, the study presents a number of limitations. First, it is important to recognise the drawbacks of animal models within the context of human-related disorders. Whilst there are overlapping behaviours and traits attributable to mice and humans in response to common precipitants, there is still an innate inconsistency comparing two different species: animal cannot be considered miniature human beings. Second, we used gene expression profiles from two different studies. While this allowed us to integrate and cross-validate results in independent samples, this means our analyses were subject to multiple testing. We did use statistically stringent correction thresholds but an inflation of false positives remains possible. Third, changes in gene expression may also be indicative of exposure to physiological changes induced by UCMS rather than the development of anhedonia or other features of depression-like phenotypes.

Finally, there have been concerns that the UCMS paradigm is difficult to replicate consistently across studies, and that combining data from two such studies, as has been done here, should be done with caution [77]. However, a recent literature review of over 60 research groups, highlighted that UCMS can induce antidepressant reversible, depressive-like behaviours, in rodents under appropriate conditions [78]. The authors did, though, describe a genuine occasional phenomenon wherein UCMS produced significant, but opposite, effects.

## Conclusion

In conclusion, an adverse environment produces stress-related differences at a genomic level, more prevalent in certain mouse strains. These differences involve several candidate genes and gene networks that favour the contribution of neurogenesis and inflammation in the pathology of Major Depressive Disorder.

## Additional files

Additional file 1
**Figure S1.** Graphs showing the correlation scores of average gene expression and overall connectivity between the two data sets. The plots show significant correlation values which are positive in all cases suggesting that the two datasets are comparable. Miller et al., report that data for Connectivity is traditionally less correlated than for Expression but all instances show a positive and significant correlation.

Additional file 2
**Figure S2.** The module labels in the BALB/cJ - C57BL/6J study still group in the GENDEP dataset showing good module preservation.

Additional file 3
**Figure S3.** PLS scores and weights. Figure A, shows scores plot with the observations coloured according to the key below the plot. Figure B shows weights plot for the variables. As was seen in the PCA scores plot, there is strong separation between the samples derived from the different strains in the first components scores (t[1]) and between the different treatments in the second components scores (t[2]).
