# Supplementary figures and images for: Pervasive and opposing effects of Unpredictable Chronic Mild Stress (UCMS) on hippocampal gene expression in BALB/cJ and C57BL/6J mouse strains
Source: BMC Genomics. 2015 Apr 3;16(1):262. doi: 10.1186/s12864-015-1431-6 (PMC4412144; doi:10.1186/s12864-015-1431-6)

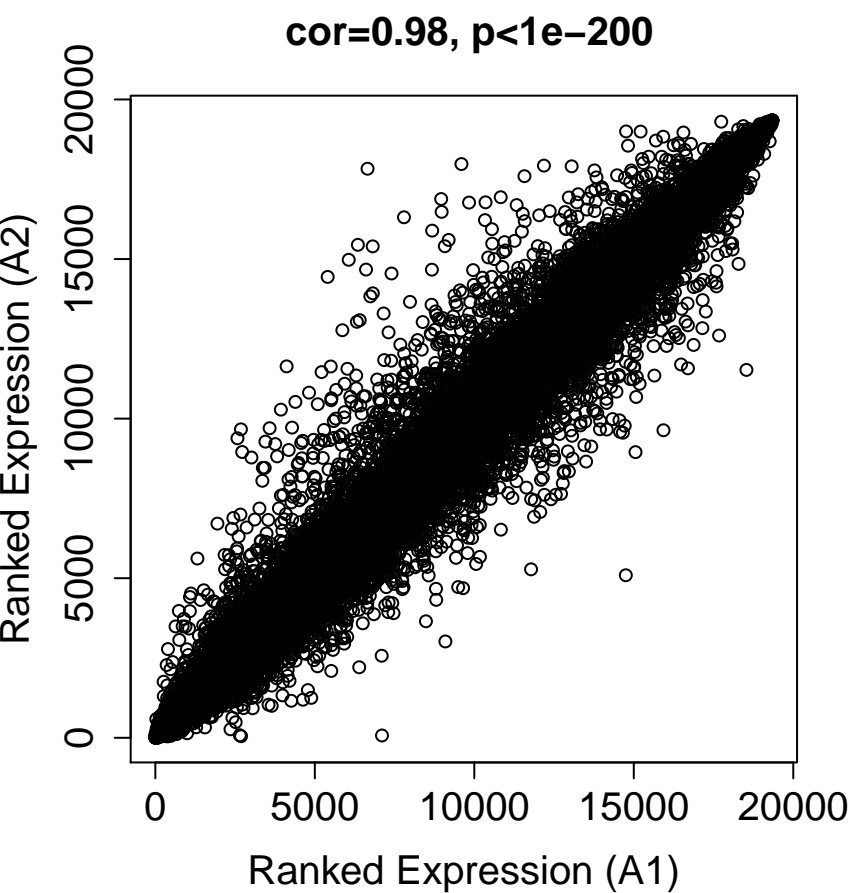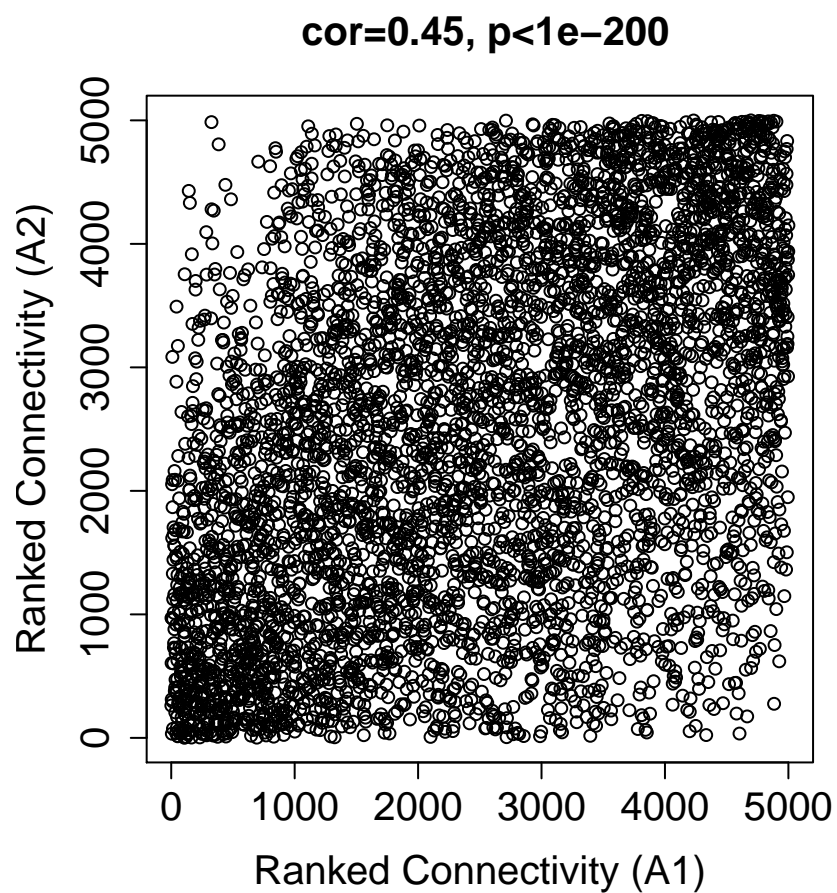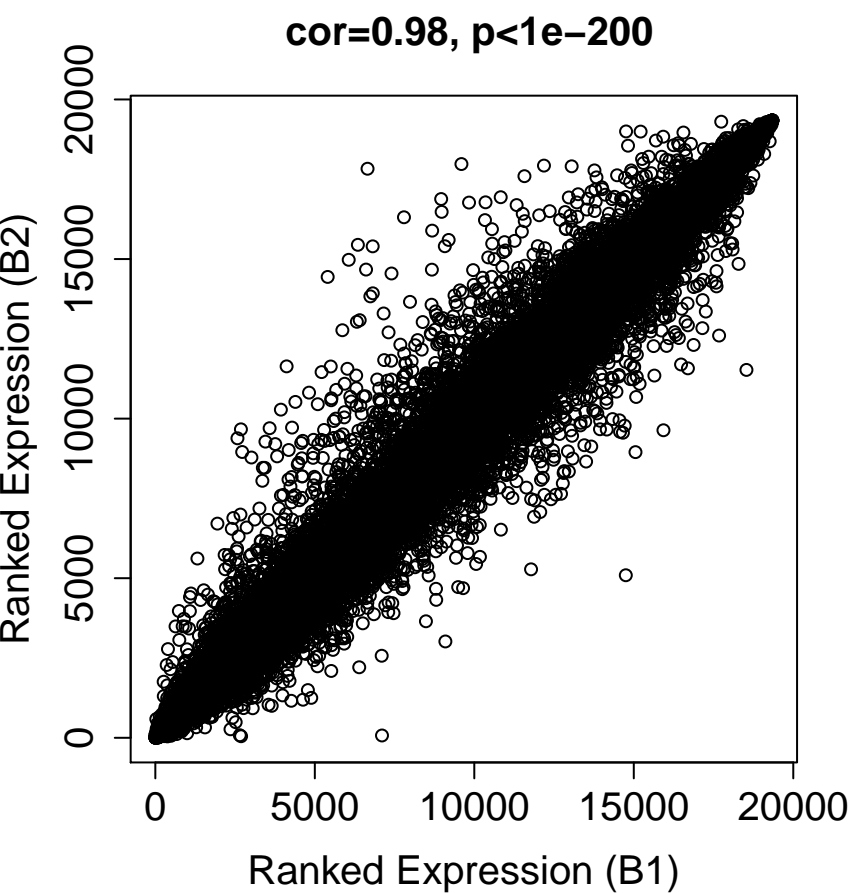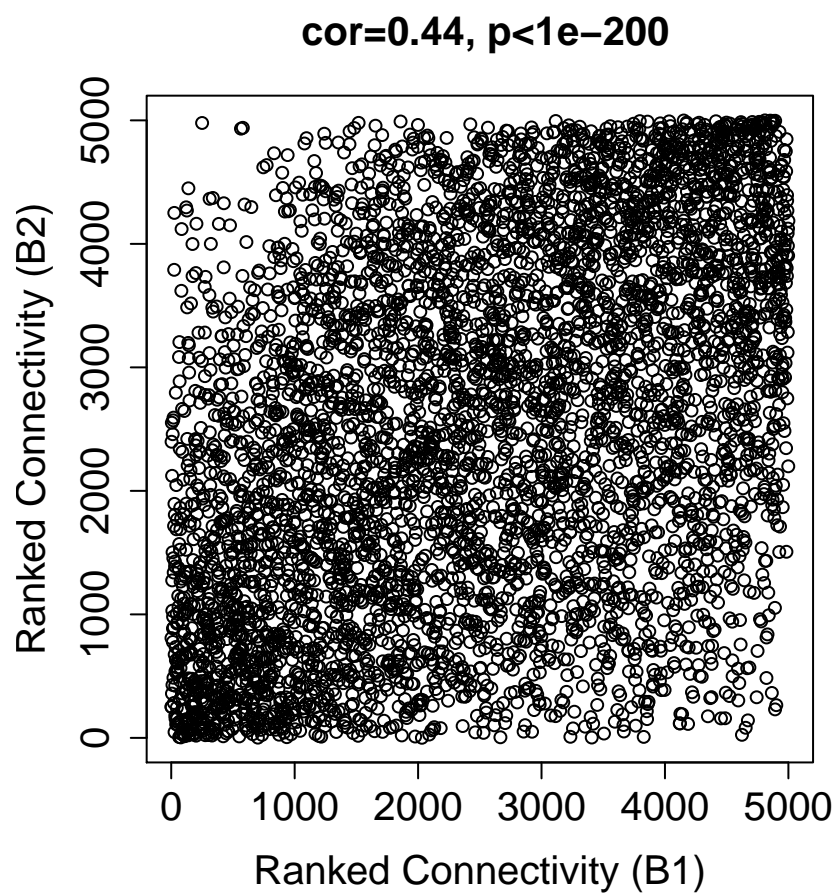

Supplement: Additional file 1 — Figure S1. Graphs showing the correlation scores of average gene expression and overall connectivity between the two data sets. The plots show significant correlation values which are positive in all cases suggesting that the two datasets are comparable. Miller et al., report that data for Connectivity is traditionally less correlated than for Expression but all instances show a positive and significant correlation. [file 12864_2015_1431_MOESM1_ESM.pdf]

Gene dendrogram and module colors (A1)

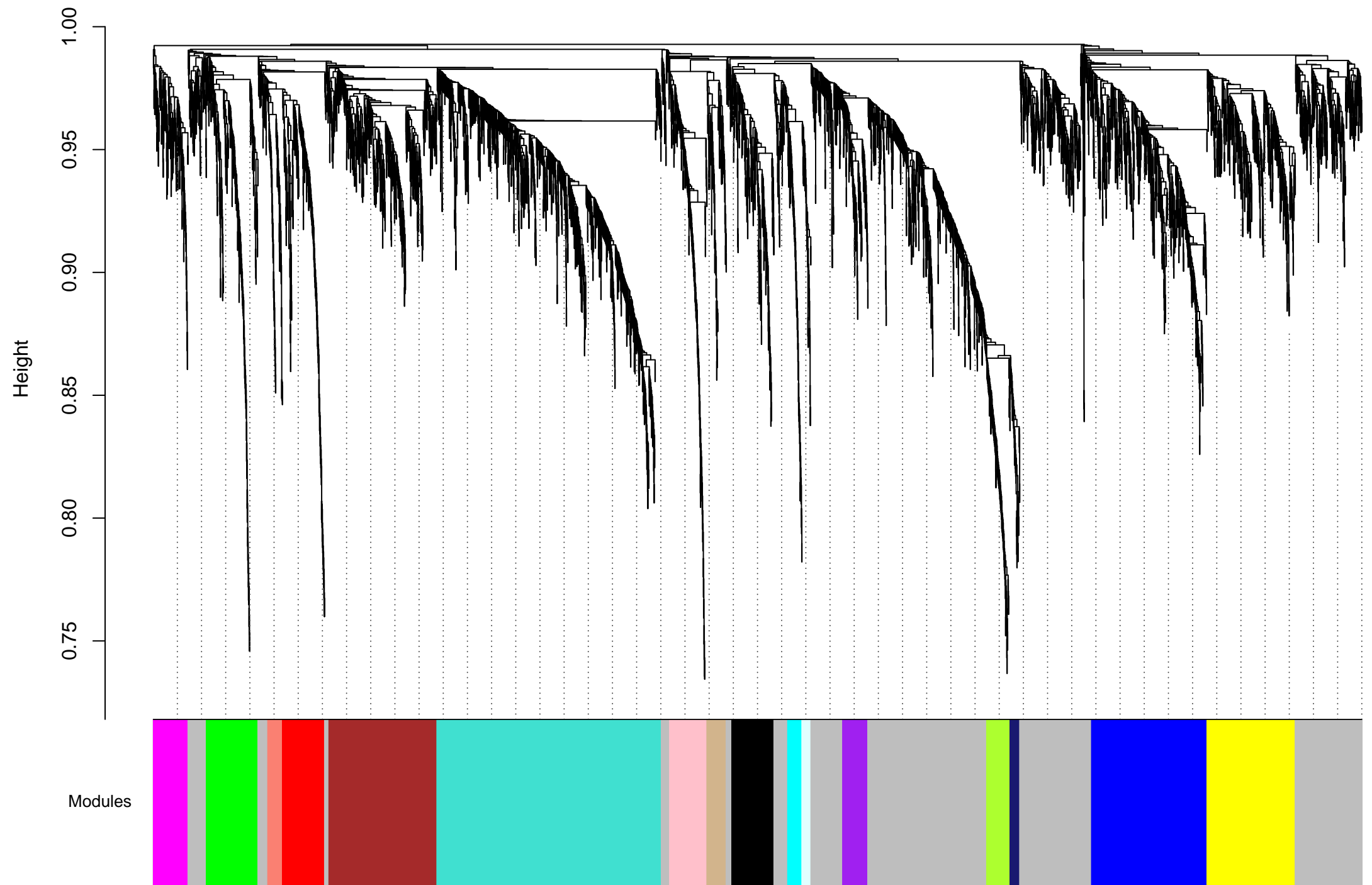

Gene dendrogram and module colors (A2)

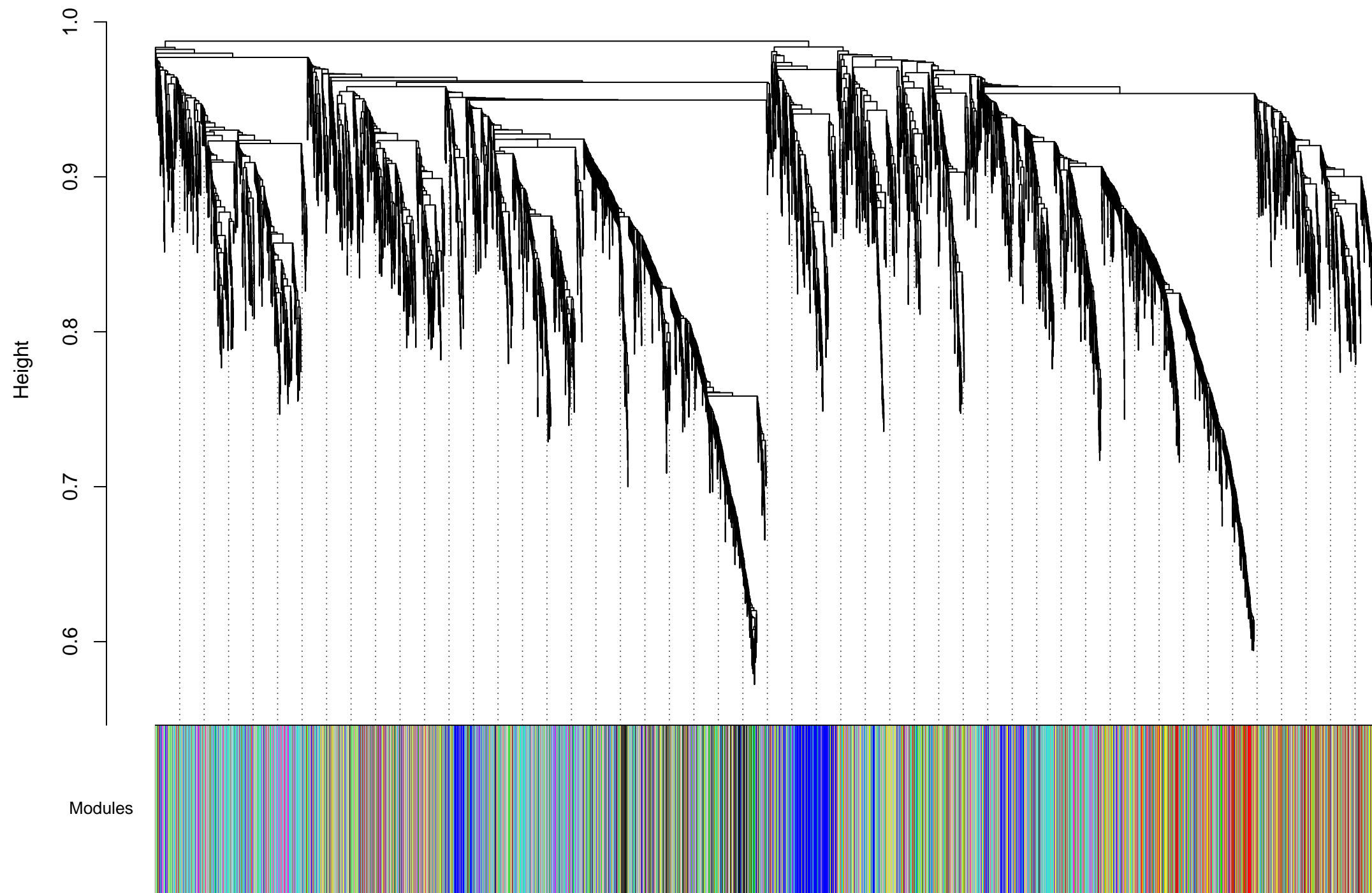

Supplement: Additional file 2 — Figure S2. The module labels in the BALB/cJ - C57BL/6J study still group in the GENDEP dataset showing good module preservation. [file 12864_2015_1431_MOESM2_ESM.pdf]

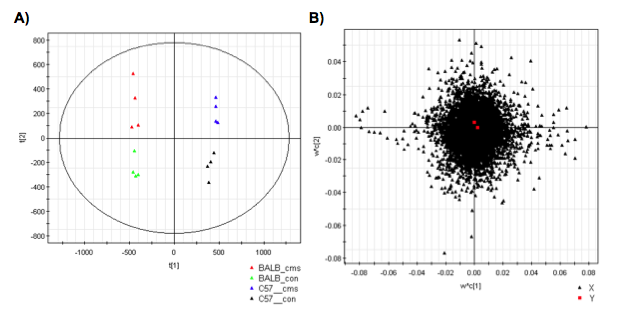

Supplement: Additional file 3 — Figure S3. PLS scores and weights. Figure A, shows scores plot with the observations coloured according to the key below the plot. Figure B shows weights plot for the variables. As was seen in the PCA scores plot, there is strong separation between the samples derived from the different strains in the first components scores (t[1]) and between the different treatments in the second components scores (t[2]). [file 12864_2015_1431_MOESM3_ESM.png]
